# Identifying the mechanism for superdiffusivity in mouse fibroblast motility

**DOI:** 10.1371/journal.pcbi.1006732

**Published:** 2019-02-14

**Authors:** Giuseppe Passucci, Megan E. Brasch, James H. Henderson, Vasily Zaburdaev, M. Lisa Manning

**Affiliations:** 1 Physics Department, Syracuse University, Syracuse, New York, United States of America; 2 Biomedical and Chemical Engineering Department, Syracuse University, Syracuse, New York, United States of America; 3 Syracuse Biomaterials Institute, Syracuse University, Syracuse, New York, United States of America; 4 Institute of Supercomputing Technologies, Lobachevsky State University of Nizhny Novgorod, Nizhny Novgorod, Russia; 5 Department of Biology, Friedrich-Alexander-Universität Erlangen-Nürnberg, Erlangen, Germany; University of Illinois at Urbana-Champaign, UNITED STATES

## Abstract

We seek to characterize the motility of mouse fibroblasts on 2D substrates. Utilizing automated tracking techniques, we find that cell trajectories are super-diffusive, where displacements scale faster than *t*^1/2^ in all directions. Two mechanisms have been proposed to explain such statistics in other cell types: run and tumble behavior with Lévy-distributed run times, and ensembles of cells with heterogeneous speed and rotational noise. We develop an automated toolkit that directly compares cell trajectories to the predictions of each model and demonstrate that ensemble-averaged quantities such as the mean-squared displacements and velocity autocorrelation functions are equally well-fit by either model. However, neither model correctly captures the short-timescale behavior quantified by the displacement probability distribution or the turning angle distribution. We develop a hybrid model that includes both run and tumble behavior and heterogeneous noise during the runs, which correctly matches the short-timescale behaviors and indicates that the run times are not Lévy distributed. The analysis tools developed here should be broadly useful for distinguishing between mechanisms for superdiffusivity in other cells types and environments.

## Author summary

Cells must move through their environment in many different biological processes, from wound healing to cancer invasion to the development of an embryo. There are different ways for cells to explore the physical space around them—ranging from moving along a straight path at constant speed to executing a random walk where the cell changes direction at every time point. Understanding what mechanisms are driving motility patterns in different cell types is important for identifying possible treatments for disease. We found that mouse fibroblast cells moving on a two-dimensional substrate were super-diffusive, meaning that they were able to cover distance faster than a random walk but not as fast as a straight walk. Traditional analysis of cell trajectories was not well-suited to distinguish between different possible mechanisms for super-diffusivity, so we developed a new tool to examine cell trajectories and distinguish between mechanisms. We found that mouse fibroblasts were super-diffusive due to a combination of large fluctuations in speed and “run-and-tumble” behavior, where cells move in a straight line for a while before changing direction rapidly. We expect this tool to be useful for analyzing motion in many other cell types.

## Introduction

Cell motility is an integral part of biological processes such as morphogenesis [1], wound healing [2], and cancer invasion [3]. But what are the rules that govern how cells move? Cell migration involves a multitude of organelles and signaling pathways [4] and therefore a fruitful, bottom-up approach studies correlations between cell motion and sub-cellular processes that govern motility, including surface interactions [5], integrin signaling pathways [6], or formation of focal adhesions [7].

An alternate approach with recent successes is to develop simple models at the cellular scale that can help identify a coarse-grained set of rules that govern cell migration in specific cell types. One such class of models, composed of self-propelled (SPP) or active Brownian particles [8] has been used to make predictions about the motion of biological cells in many contexts, including density fluctuations [9], formation of bacterial colonies [10], and both confined [11], and expanding monolayers [12].

These SPP models represent each cell as a particle that moves by generating active force on a substrate, which acts along a specified vector $\hat{\theta }$. Therefore, the parameters for the model specify both the magnitude of the force as well as how the orientation of the force changes with time. Given the ubiquity and usefulness of these models, one would like to have a standard framework for extracting these parameters from experimental data for all trajectories. In the past this has often been accomplished by analyzing ensemble-averaged features of cell trajectories.

One such quantity is the time averaged mean-squared displacement (MSD), which is the squared displacement between positions $\vec{r}\left(t\right)$ and $\vec{r}\left(t+dt\right)$ averaged over all starting times *t* and the ensemble of trajectories. This yields the MSD as a function of timescale, 〈(*r*(*t* + *dt*)) − *r*(*t*))^2^〉 ∝ *dt*^*α*^. Ballistic motion, which corresponds to a cell moving in a straight line at constant speed, corresponds to *α* = 2. Diffusive motion, where a cell executes a random walk with no time correlation in orientation, corresponds to *α* = 1. In non-active matter at low densities, thermal fluctuations generically induce diffusive behavior at long timescales. In contrast, many cell types, including T-cells [13], Hydra cells [14], breast carcinoma cells [15], and swarming bacteria [16] display super-diffusive dynamics, defined as trajectories with a MSD exponent between 1 < *α* < 2.

Several authors have proposed explanations for why super-diffusive migration might be beneficial in biological systems. For example, super-diffusive trajectories are well known for being the optimal search strategy for randomly placed sparse targets [17, 18], and have been found in animal foraging and migration patterns in jellyfish [19], albatross, and bumblebees [20]. In the context of cell biology, superdiffusive migration implies that cells are covering new areas more quickly than they would if they were executing a simple random walk.

Although super-diffusive dynamics are commonly observed in *in vitro* experiments, the fundamental mechanism that generates anomalous diffusion in cell trajectories remains unclear. Pinpointing the mechanism would allow biology researchers to better isolate the signaling pathways that govern these processes.

Although one might think that simply including the effects of persistent active forces generated by cells would change the long-time behavior, it turns out that standard self-propelled particle models exhibit a fairly sharp crossover from ballistic to diffusive motion, with no extended superdiffusive regime. Since SPP models are commonly used to model cells and superdiffusive dynamics are commonly observed in experiments, we would like to identify the mechanism generating superdiffusitivity to improve the ability of these models to capture cellular phenomena.

Standard SPP models include smoothly varying persistent random walkers and standard run-and-tumble particles (RTP) [21]. Persistent random walkers obey the following equations of motion for the cell center of mass *r*_*i*_ and the orientation angle *θ*_*i*_:

$$\partial_{t}\vec{r_{i}}=v_{0}{\hat{\theta }}_{i},$$ (1)

$$\partial_{t}\theta_{i}=\eta \left(t\right),$$ (2)

where *η*(*t*) is a Gaussian white noise (〈*η*(*t*)*η*(*t*′)〉 = 2*D*_*r*_ *δ*(*t* − *t*′)). In a standard persistent random walk, the speed *v*_0_ and the rotational diffusion coefficient *D*_*r*_, which controls the strength of fluctuations in orientation, are constant. In a standard run-and-tumble model, particles are ballistic during runs, ∂_*t*_*θ*_*i*_ = 0, followed by tumbling events where large changes in orientation occur. Variations of run-and-tumble models are characterized by the distribution of times particles remain in the run state.

Two different classes of modifications to SPP models have been highlighted as being able to generate super-diffusive behavior on long timescales. The first modification is a heterogeneous speed model, which draws rotational diffusion coefficients and particle speeds from distributions [15, 22]. While persistent random walk models transition from ballistic to diffusive behavior at one characteristic timescale, heterogeneous speed models possess a heterogeneous distribution of crossover timescales, which generates an MSD with a broad superdiffusive regime, though the system becomes diffusive on timescales longer than $1/D_{r}^{min}$.

The second modification is a Lévy walk model, which is a run-and-tumble model where particles have power law distributed run times:

$$P\left(\tau \right)=\frac{\mu }{\tau_{o}{\left(1+\tau /\tau_{o}\right)}^{1+\mu }},$$ (3)

$$\left\langle \tau \right\rangle =\frac{\tau_{o}}{\mu -1},$$ (4)

with *P*(*τ*) the distribution of run times with mean < *τ* > for *μ* > 1. [23]. In contrast to the heterogeneous SPP model, super-diffusivity generated by Lévy walks is not transient, so that the long-time MSD scaling exponent is constant: *MSD* ∝ *dt*^3−*μ*^.

So which of these models is the “right” one for a given cell type? By analyzing ensemble-averaged statistics such as the MSD and the velocity autocorrelation function (VACF), one group of researchers was able to show that heterogeneous motility models matched data from breast cancer carcinoma cells [15]. This model, based on an autoregressive process (AR-1), uses a Bayesian inference method to extract activity and persistence from cell trajectories. However, these quantities do not directly correspond to physical quantities such as cell speed or rotational diffusion. Effects of cell heterogeneity were also explored in human fibrosarcoma cells by Wu et al., where the authors show that these effects are sufficient to explain non-Gaussian velocity distributions [24], similar to those we observe in mouse fibroblast cells. The authors also investigate anisotropic contributions, modeling 3D human fibrosarcoma trajectories with a 3D anisotropic persistent random walk.

Differentiating between inherently anisotropic behavior and cell response to external cues such as chemotaxis is another difficult problem, investigated in T cells by Banigan et al. using a unique model that features a mix of passive Brownian particles and persistent random walkers [25]. Other efforts evaluated a different ensemble-averaged quantity, the probability displacement distribution, and used that data to suggest that T-cells were undergoing generalized Lévy walks [13]. We would like to better understand whether these ensemble-averaged quantities are in fact a unique identifier of the underlying mechanism for superdiffusivity. Moreover, we also seek to develop a systematic procedure for using experimental data to constrain both the appropriate mechanism and the optimal model parameters for a specific subtype. To this end, we use automated tracking software to analyze over 1000 mouse fibroblast trajectories and, using the work of Metzner and colleagues as inspiration, extract parameters for a generalized model based on persistent random walkers. We demonstrate that some ensemble-averaged statistics, such as the MSD and VACF, can not distinguish between mechanisms for superdiffusivity.

In order to better distinguish, we begin with a very general model for cell dynamics. Although standard SPP models have only two fit parameters, average cell speed *v*_0_ and average rotational noise *D*_*r*_, in principal a generalized SPP model could have arbitrary distributions for cell speed *P*(*v*_0_) and rotational diffusion *P*(*D*_*r*_) with arbitrary correlations between them. The heterogeneity motility model from [15] is the limit of such a model with Gaussian-distributed *P*(*v*_0_) and *P*(*D*_*r*_), while a standard Lévy walk is the limit with a constant *v*_0_ and a specialized bimodal *P*(*D*_*r*_). Generalized Lévy walks such as those studied in [13] have additional parameters. Because this is such a large parameter space, we first constrain the functional form of these distributions using specific features of single cell trajectory statistics. We find that the mouse fibroblast data are consistent with run-and-tumble dynamics but the run times are not power-law distributed, confirming that in mouse fibroblasts the mechanism for superdiffusivity is heterogeneous dynamics and not Lévy walk statistics. The toolkit we have developed here should be useful for pinpointing the origin of superdiffusivity in many other cell types.

## Materials and methods

### Mouse fibroblast cell culture

Cell motility data was collected from C3H10T1/2 mouse fibroblast cells (ATCC). Although the cells were cultured on gold-coated shape memory polymer substrates, which in principle can be programmed to form anisotropic nanowrinkles [26], all of the data in this manuscript is from cells cultured on “control” substrates that remain flat throughout the entire experiment, as our goal is to characterize the origin of superdiffusivity in this most simple case. While the data used in this manuscript are from an experimental protocol with a temperature shift, Baker et al. saw very similar superdiffusive trajectories in systems with no temperature changes [27], indicating that the superdiffusivity we observe here was not generated by or dependent on temperature changes. Future work will analyze behavior on more complicated wrinkled or transitioning substrates. Cell nuclei were labeled with Hoechst dye and cell motility imaged by time-lapse microscopy. The resultant image stacks were analyzed using the ACT*IV*E image analysis package to track nuclei centers-of-mass [27]. See S1 Text for more information on substrate preparation.

### Cell trajectory analysis and particle simulations

**Fig 1:**
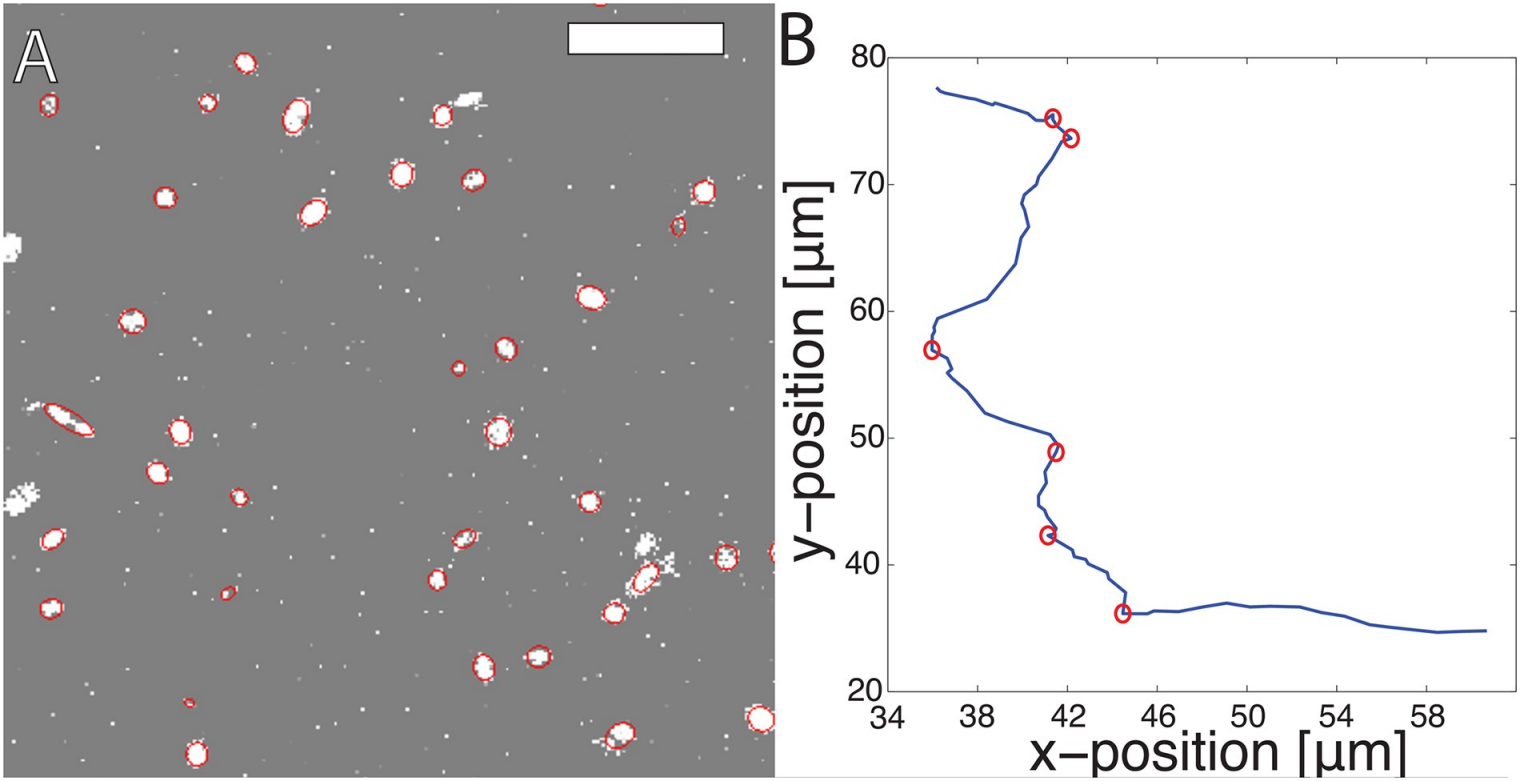
Experimental cell environment with typical trajectory. (A) An example image of nuclei stained with Hoescht dye. The scale bar is 500 *μm*. These images were processed using the ACT*IV*E image analysis package to track nuclei centers-of-mass [27], with overlaid best-fit ellipses. (B) A typical cell trajectory with tumbling events indicated by red circles, as identified by the 2D Canny edge detection algorithm.

Cell motility was characterized using statistical analysis of cell nuclei trajectories, including MSD, VACF and displacement probability distributions. Tumbling events were identified with a one dimensional Canny edge detection algorithm, as shown in Fig 1. This algorithm takes a time series of changes in orientation and classifies each timestep as either a “run” or “tumble.” Additional details on cell trajectory analysis can be found in S1 Text.

### Active particle simulations

This manuscript focuses on two different models for non-interacting active particles. The first model is a Lévy walk with constant particle speed *v*_0_ at all timesteps. Particles execute ballistic runs with zero rotational noise for times *τ* drawn from the distribution in Eq 3 and a mean run time 〈*τ*〉 given by Eq 4.

The generalized SPP model has particles which follow the equations of motion seen in Eqs 1 and 2, however the parameters for each model are not constant in time. A particle is initialized with a random orientation and assigned an initial speed *v*_0_ and rotational diffusion *D*_*r*_ drawn from distributions $P\left(v_{0}\right)=\frac{|v_{0}|}{\sigma_{v}^{2}}e^{-\frac{{\left(v_{0}-\mu_{v}\right)}^{2}}{\sigma_{v}^{2}}}$ and $P\left(D_{r}\right)=\frac{1}{\sqrt{\pi \sigma_{D}^{2}}}e^{-\frac{{\left(D_{r}-\mu_{D}\right)}^{2}}{\sigma_{D}^{2}}}$. To account for possible correlations between the speed and rotational diffusion variables in our model, we utilize a copula modeling method [28]. First, we sample a bivariate normal distribution with a covariance matrix given by $\Sigma =\left[\begin{array}{cc} 1 & p\\ p & 1 \end{array}\right]$, where *p* = 0 indicates no correlation and *p* = ±1 indicates full positive (negative) correlation. Then we use the standard method of inverse cumulative distribution functions to transform the marginal distributions into the distributions *P*(*v*_0_) and *P*(*D*_*r*_) listed above. This results in a set of variables with a correlation between them parameterized by *p*, and also with the desired marginal distributions.

Following sampling *v*_0_ and *D*_*r*_, we evolve the particle position and orientation for a time *τ* drawn from $P\left(\tau \right)=\frac{1}{\tau_{0}}e^{\tau /\tau_{0}}$, where *τ*_0_ is the mean run time determined by experimental data. The particle then undergoes a tumbling event across one time step where *D*_*r*_ = 2*π*, where the value of rotational diffusion is chosen to approximate an event where the orientation is completely randomized. After the tumble a new *v*_0_ and *D*_*r*_ are assigned until the next tumbling event. In contrast to a Lévy walk or standard SPP model, motility parameters are varied in time to replicate the variations and changes in a biological environment.

For both models, particle trajectories are constructed by numerically integrating the equations of motion using a simple Euler scheme with a timestep *dt* = 0.1. For fitting purposes, we choose the natural timescale in our simulations equal to four minutes in experiments, which is the time between frame captures. In addition, we use the averaged goodness-of-fit of model MSD, VACF and displacement probability distributions compared to that of mouse fibroblast trajectories to determine optimal model parameters, shown in Table 1 and discussed later in the text.

**Table 1:** Model parameters for the Lévy walk (LW) and generalized self-propelled particle (Gen. SPP) models as well as parameters derived from microscopic statistics. Speed is in units of *μ*m/min, rotational diffusion in units of sec^−1^ and run time in units of hours.

	*μ*	*v*_0_	*μ*_*D*_	*σ*_*D*_	*μ*_*v*_	*σ*_*v*_	*p*	*τ*_0_
LW	1.4	1.7	-	-	-	-	-	10
Gen. SPP	-	-	0.09	0.002	1.2	0.8	0	10
Microscopic	1.5	1	0.11	0.001	1	0.7	0	11

Finally, we note the VACF for experimental data shows a sharp dropoff across one frame due to errors in reconstructing the nuclei centers caused by imaging noise and fluctuations in dye intensity. To replicate this feature we incorporate positional noise into both models through small Gaussian fluctutations. After particle trajectories are constructed, each position is changed by a vector $\delta \vec{r}=dr\hat{\phi }$, where *dr* is drawn from a Gaussian distribution of variable width Δ and the direction $\hat{\phi }$ is chosen randomly from the unit circle. This replicates experimental error in reconstructing cell positions, and allows our model trajectories to match the mouse fibroblast data.

## Results

### Experimentally observed ensemble-averaged quantities are well fit by several existing models

**Fig 2:**
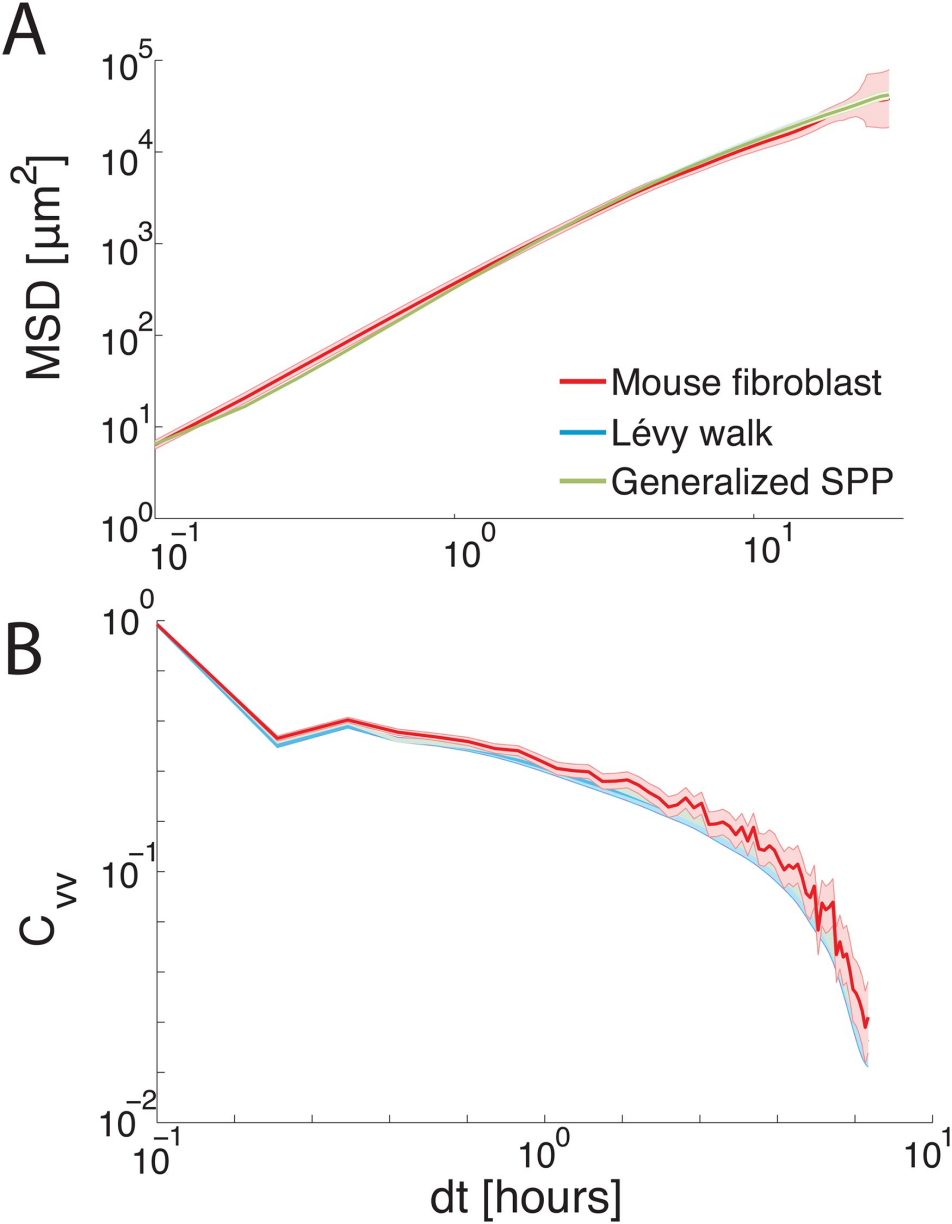
Ensemble-averaged statistics are similar across models. All of the proposed superdiffusive models are capable of capturing ensemble-averaged mouse fibroblast statistics. (A) Mean-squared displacements for mouse fibroblast cells, shown in red, as well as a Lévy walk and a generalized SPP model. Both models are able to match the mouse fibroblast MSD within the margin of error. (B) The velocity auto-correlation function *C*_*vv*_ as a function of time *dt*. There is a sharp decrease in the VACF across the first frame, due to error in reconstructing the nuclei centers-of-mass generated by imaging noise and fluctuations in dye intensity. At the largest timescales, each bin corresponds to fewer events and so error bars become large. In addition, adding positional error to simulation trajectories to match the initial dropoff in the VACF causes significant fluctuations at larger timescales.

Previous reports have compared models to experimental data using ensemble-averaged statistics to confirm model validity such as the MSD and the VACF. Therefore, our first goal is to determine whether one of the existing models for explaining superdiffusive cell trajectories is a better fit to the experimental MSD and VACF data, shown by the red lines in Fig 2.

For comparison, we simulate a Lévy walk model with dynamics given by Eqs 3 and 4, as well as a generalized SPP with no Lévy-walk behavior, described in more detail below. With the best-fit parameters, we find that both models match the data equally well. As shown in Fig 2(B), the velocity autocorrelation function exhibits a sharp decrease after the first frame window, due to errors that we make in reconstructing the nuclei center of mass caused by imaging noise and fluctuations in the dye intensity. Therefore, we add an additional term to the model that shifts the particle position by a Gaussian distributed variable with zero-mean and variance Δ^2^ to account for this effect.

**Fig 3:**
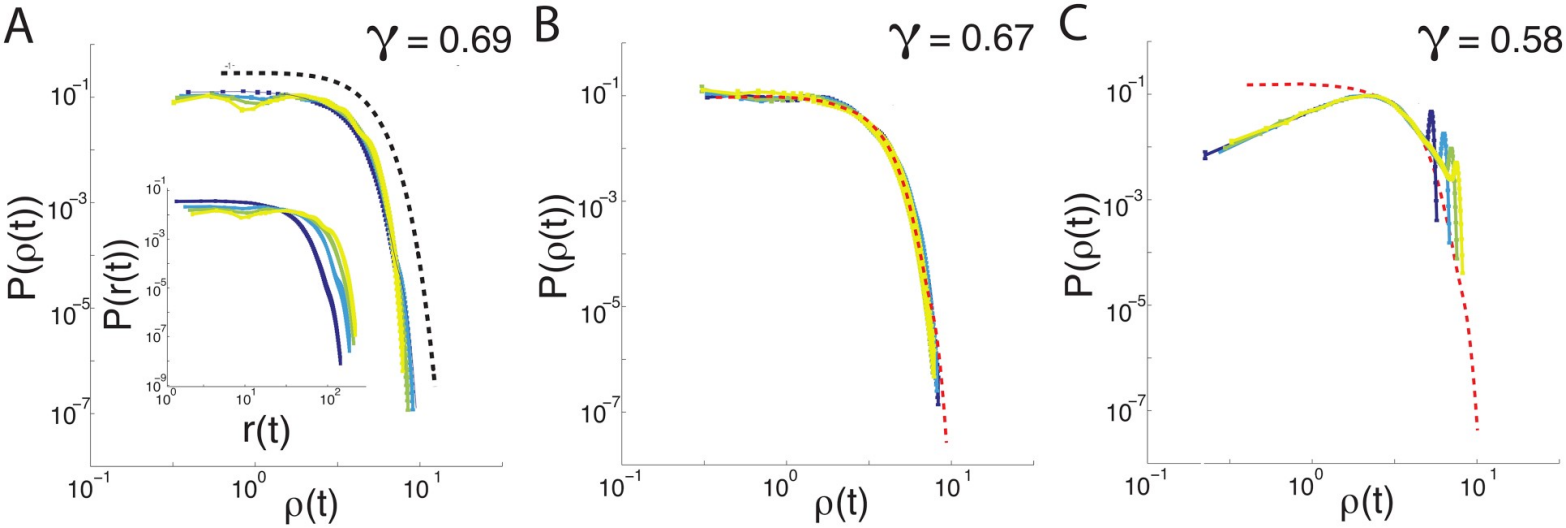
Displacement probability distributions collapse at similar scaling exponents. Displacement probability distribution *P*(*ρ*), where *ρ*(*t*) is the scaled displacement $\frac{r(t)}{t^{\gamma }}$, for the value of *γ* that best collapses the data, for (A) Mouse fibroblast cells, (B) generalized SPP model and (C) Lévy walk, with colors representing 4 binned timescales from blue (small) to yellow (large) at times *t* = 5, 10, 15 and 20 hours. Mouse fibroblast $\tilde{P}\left(\rho \right)$ is shown as a dashed red line in (B, C) for comparison with each model, showing that only the generalized SPP model is consistent with the observed data. Collapsed mouse fibroblast distributions are fit to a Gaussian (A) shown as a dashed black line, artificially shifted for visibility. See Fig 4 for a characterization of different *γ* values for each model, characterized by *χ*^2^.

**Fig 4:**
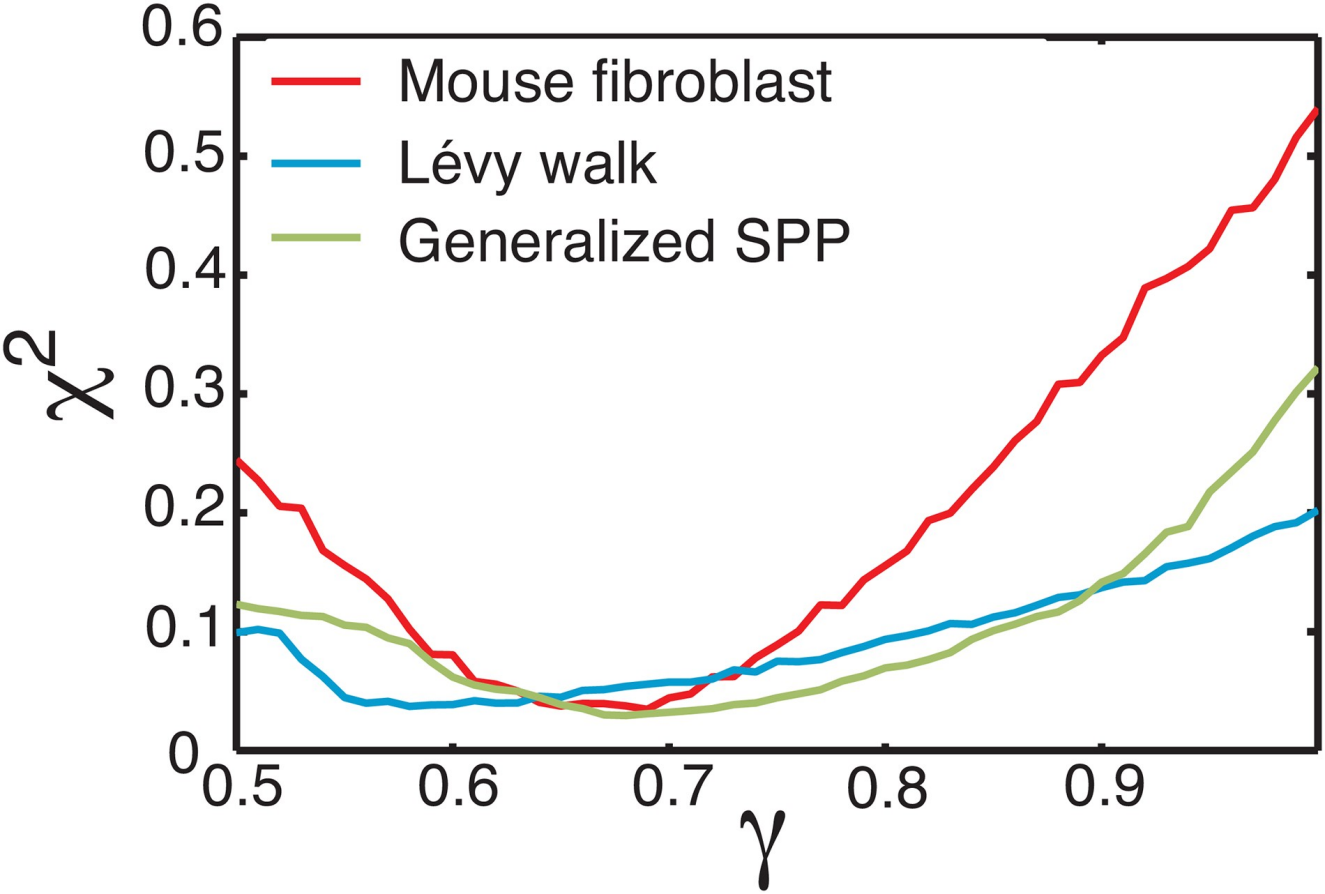
*χ*^2^ of distribution collapse begins to differentiate models. Goodness-of-fit (*χ*^2^) as a function of scaling exponent *γ*. The value of *γ* that best collapses each data set minimizes the *χ*^2^ goodness of fit between the *P*(*ρ*(*t*)) calculated at each timescale. Experimental data all collapse at a value of 0.5 < *γ* < 1, consistent with a superdiffusive MSD.

While the mean-squared displacement and velocity auto-correlation function are standard metrics for characterizing ensembles of trajectories, they may not be ideal for studying systems with superdiffusion. In an investigation of the Lévy walk properties of T-cells, Harris et al. study a quantity that reveals structures on shorter timescales: the probability for a cell to be at a displacement *r*(*t*) at time *t* [13]. They suggest that generalized Levy walks can be distinguished in part by collapsing these probability distributions with rescaled displacements $\rho \left(t\right)=\frac{r(t)}{t^{\gamma }}$, with *γ* significantly larger than the value of 1/2 expected for a persistent random walk. In their initial work characterizing Lévy walks, Harris and colleagues considered a wide range of Lévy walks as well as several other random walk processes, and finding the best match for T-cell trajectories was to a generalized Lévy walk. As seen in Fig 3, we find that the mouse-fibroblast data does collapse, with the best fit exponent *γ* = 0.69 ± 0.02 as shown in Fig 4. The best-fit standard Levy walk model collapses at *γ* = 0.58 ± 0.03, which is above the value expected for a persistent random walk but still lower than *γ* for mouse fibroblast cells. Importantly, the best-fit generalized SPP model also collapses at a similar value of *γ* = 0.67 ± 0.03, suggesting that such a collapse is not sufficient to uniquely identify Lévy walks as a mechanism for superdiffusivity.

Moreover, the functional form of the displacement probability distribution (PDF) *P*(*r*(*t*)) provides additional information. It is well-fit by a Gaussian curve, shown as an offset dashed black line in Fig 3(A) and 3, and a Fig 3(B) shows that non-Lévy version of the generalized SPP model also matches the shape of mouse fibroblast *P*(*r*(*t*)) very well. In contrast, Fig 3(C) shows that *P*(*r*(*t*)) for the best-fit standard Lévy walk model has a very different functional form, due to ballistic runs over relatively large distances. However, this mis-fit in the functional form does not rule out Levy walks as a possible mechanism, as it could be corrected by considering a generalized Lévy Walk with more parameters [13]. To truly distinguish between the two mechanisms, we need access to more granular details about the individual cell trajectories.

### Numerical models are better constrained by single-cell trajectory data

**Fig 5:**
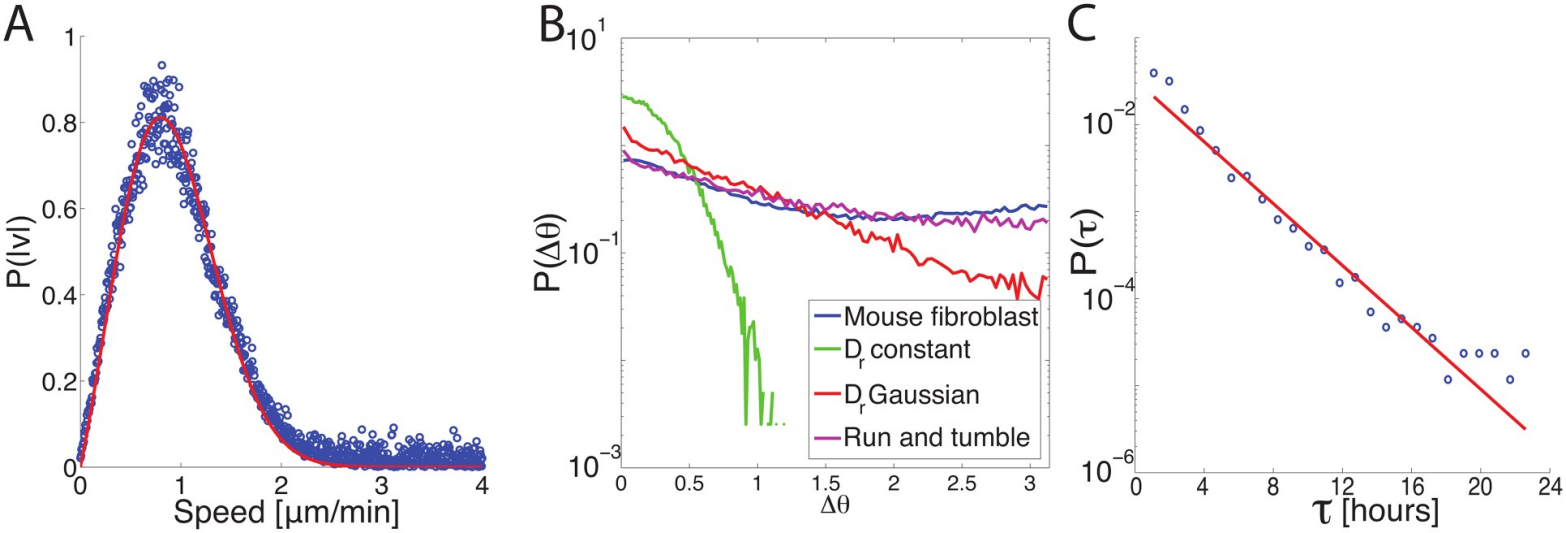
Distributions of small-scale parameters support generalized SPP. (A) Distribution of mouse fibroblast instantaneous speeds calculated from cell nuclei center-of-mass displacement between image capture events (blue). The red line is a fit to the form $P\left(v_{0}\right)=\frac{|v_{0}|}{\sigma_{v}^{2}}e^{-\frac{{\left(v_{0}-\mu_{v}\right)}^{2}}{\sigma_{v}^{2}}}$, which is the distribution of speeds expected for a Gaussian distribution of velocities. (B) Distribution of turning angles of mouse fibroblast trajectories (blue), SPP models with constant *D*_*r*_ (green), Gaussian distributed *D*_*r*_ (red), and a run-and-tumble model with Gaussian distributed *D*_*r*_ during runs and exponentially distributed tumbling events. The distribution of rotational diffusion constants is the same in both heterogeneous cases to highlight the effect of incorporating tumbling events into the system. (C) Run-time distribution for mouse fibroblast cells (blue) is well fit by an exponential distribution (red).

We next study single-cell trajectories. A generalized SPP model with arbitrary distributions for *P*(*v*_0_) and *P*(*D*_*r*_) has an infinite number of parameters that we could never hope to constrain. As a first step to simplifying our model we constrain functional form of these distributions using experimental data through microscopic statistics, such as velocity and run-time distributions, calculated from single-cell trajectories. This is in contrast to ensemble and time-averaged macroscopic statistics such as MSD and VACF. As shown in Fig 5(A), we first construct a distribution of cell speeds, determined from the magnitudes of the displacement of nuclei centers-of-mass between image capture events. Our experimental data is consistent with a Gaussian distribution of cell velocities, or equivalently, a distribution of cell speeds of the form $P\left(v_{0}\right)=\frac{|v_{0}|}{\sigma_{v}^{2}}e^{-\frac{{\left(v_{0}-\mu_{v}\right)}^{2}}{\sigma_{v}^{2}}}$, where *μ*_*v*_ and *σ*_*v*_ are the mean and standard deviation, respectively, with estimates shown in Table 1. Therefore, we use this functional form in our generalized model. Next we estimate a distribution *P*(*D*_*r*_) of rotational diffusion constants (*D*_*r*_) from the distribution of turning angles, shown in Fig 5(B). Simple active Brownian systems with a single value of *D*_*r*_ will generate a Gaussian distribution of turning angles [21]. A Gaussian distribution of rotational noise broadens this distribution significantly. One can show the expected turning angle distribution in this case is a modified Bessel function of the second kind with an exponential tail, consistent with the numerical simulation data given by the red line in Fig 5(B). We were unable to match the mouse fibroblast turning angle distribution, which is given by the blue line in Fig 5(B) and has significant weight as the largest values of Δ*θ*, with any Gaussian function for the rotational noise.

This suggests that mouse fibroblast cells may have a strongly bimodal distribution of rotational noises, further supported by intermittent run-and-tumble behavior seen in cell trajectories. We choose to capture this bimodal behavior with a noisy run-and-tumble model, where cells have a distribution $P\left(D_{r}\right)=\frac{1}{\sqrt{\pi \sigma_{D}^{2}}}e^{-\frac{{\left(D_{r}-\mu_{D}\right)}^{2}}{\sigma_{D}^{2}}}$ during runs, which are punctuated by tumbling events. Distribution parameters *μ*_*D*_ and *σ*_*D*_, shown in Table 1, can be estimated from the distribution *P*(*D*_*r*_) used to generate the run and tumble distribution of turning angles seen in Fig 5(B). In our implementation of this model we include possible arbitrary correlations between these distributions through the parameter *p*, ranging from fully correlated (*p* = 1) to anti-correlated (*p* = −1). We use the Canny algorithm described in the methods section to explicitly identify tumbling events, and the data points in Fig 5(C) show the distribution of times between such events. The red line in Fig 5(C) shows this is well-fit by an exponential distribution with *τ*_0_ ≈ 1 hour, and so in our model the distribution of run times *τ* is given by $P\left(\tau \right)=\frac{1}{\tau_{0}}e^{-\tau /\tau_{0}}$. We note that this is a strong indication that the mouse fibroblasts are not well-described by a Lévy walk model with power-law distributed run times. Specifically, although we focus here on a standard Lévy walk model with fewer parameters than the generalized model used by Harris et al. [13], it is clear that adding additional parameters to our a Lévy walk will still not generate the *P*(*τ*) we observe in mouse fibroblasts. The magenta line in Fig 5(B) shows the distribution of turning angles for a noisy run-and-tumble model with the parameters identified above.

To confirm that the model parameters we have identified are robust, and to quantify their sensitivity, we vary model parameters around the microscopically determined values and quantify how much this changes their displacement probability distributions. Specifically, we use the linear regression goodness-of-fit parameter (*R*^2^) between *P*(*r*(*t*)) for mouse fibroblast and generalized model trajectories to characterize each parameter configuration and identify a best-fit between our model and mouse fibroblast statistics [29]. Using this method we are able to capture the functional form of *P*(*r*(*t*)) very well, as shown in Fig 3. We explored several additional methods to parameterize goodness-of-fit, but because the shape of *P*(*r*(*t*)) exhibited significant fluctuations as we swept parameter space, nonlinear fitting approaches were inconsistent. Therefore we focus on the more stable linear regression results here.

**Fig 6:**
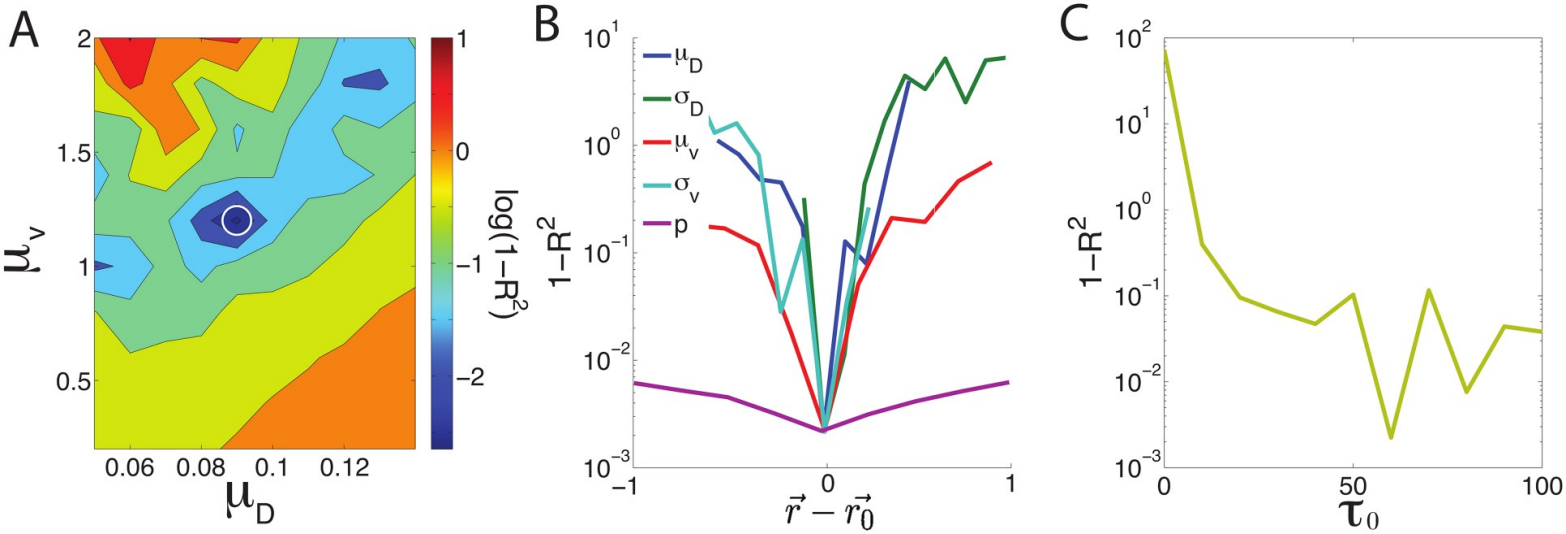
Model sensitivity calculated through exploring phase space. Sensitivity analysis examining the goodness of fit of the generalized SPP model to displacement distributions in mouse fibroblasts (1 − *R*^2^) as a function of model parameters. (A) Contour plot of *log*(1 − *R*^2^) illustrates the experimental data tightly constrains a linear combination of the mean velocity *μ*_*v*_ and mean noise *μ*_*D*_, where the minima is highlighted by a white circle. (B, C) The goodness of fit as a function of each model parameter while all others are held fixed. (B) $\vec{r_{0}}$ is the optimal coordinate in parameter space and $\vec{r}-\vec{r_{0}}$ is the distance of each parameter from its optimal value. (C) A value of *τ* smaller than ≈ 10 is inconsistent with experimental data, but data does not discriminate between larger values of *τ*.

Happily, the configuration of parameters that best matches the macroscopic *P*(*r*(*t*)), located at *μ*_*D*_ = 0.09, *σ*_*D*_ = 0.002, *μ*_*v*_ = 1.2, *σ*_*v*_ = 0.8, *p* = 0, *τ*_0_ = 10, is very similar to those identified from microscopic statistics, indicating that the model is consistent with experimental results. A construction of the dynamical matrix as a Hessian in parameter space around this minima and subsequent analysis of local eigenvectors indicates that our system is most sensitive to perturbations in the mean velocity and mean rotational noise as shown in Fig 6(A), and relatively insensitive to correlations between *D*_*r*_ and *v*_0_ parameterized by *p* (Fig 6B) as well as mean run time *τ*_0_ (Fig 6C).

## Discussion

Both Lévy walks and heterogeneous SPP models are capable of generating superdiffusive trajectories. Previous studies have focused on one model or the other in order to identify possible mechanisms for superdiffusive cell trajectories.

We show that while both types of model are equally capable of matching the large-scale ensemble averaged statistics of mouse fibroblast cells, an analysis of single cell trajectories demonstrates that Lévy walks are not consistent with this data set, despite a very good scaling collapse of the probability displacement distribution with scaling exponent *γ* > 1/2. Instead, a careful analysis of turning angle distributions suggests these mouse fibroblasts exhibit heterogeneous speeds, with noisy run-and-tumble behavior.

Because superdiffusive cells are able to cover distance faster than diffusive counterparts, it would be useful to adapt the tools developed here to study many more cell types. For example, directed cell motion is known to be a signature of invasiveness in cancer cell lines [30], and it would be interesting to know if these cell types are altering the mechanisms or timescales for superdiffusion as they become more malignant. To that end, we have created a MATLAB software package for deploying these analyses on generic data sets [31], which can be used to quantify superdiffusive dynamics and distinguish between different mechanism behavior in cells and active matter.

Another important question is whether the tumbling events seen here are cell-autonomous or generated by cell-cell interactions. On the one hand, It is possible that the run and tumble behavior is at least partially cell-autonomous, although no biochemical mechanisms for such behavior have been identified in fibroblasts. To begin to investigate this question, it would be useful to correlate tumbling events with the dynamics of sub-cellular features such as spatio-temporal distributions of focal adhesions [32], Golgi bodies [33], or actin waves [30]. This would help us to understand which signaling networks and components of motility machinery are involved in generating tumbling behavior or broad distributions of rotational diffusion. Furthermore, it might be useful to study such behavior on structured or controllable substrates [34], to tease apart the influence of environment vs. internal circuitry on controlling these timescales.

On the other hand, many cell types exhibit contact inhibition of locomotion (CIL) [35], where contact with another cell will either halt their motion or cause them to immediately recoil and begin moving in the opposite direction. It is possible that the tumbling events we see in mouse fibroblast cells are CIL events. In this work mouse fibroblast trajectories were identified from nuclei centers-of-mass, and we do not have direct observations of the cell membrane. Due to this imaging limitation, we were not able to confirm which tumbling events are associated with cell-cell contacts. This would be an interesting avenue of future research, as our cells are seeded at intermediate densities and it is possible that a significant fraction of of tumbling events are caused by cell-cell contacts. If so, this would be an interesting mechanism for generating super-diffusive behavior of a group of cells at intermediate densities, which could contribute to enhanced diffusion of moving cell fronts.

In addition to CIL, there could also be additional interactions between cells, such as alignment of motility polarization between neighbors or between cells and the underlying substrate to generate flocking-like behavior [8]. It would be interesting to explore the effect of alignment in a generalized SPP model, to see if heterogeneity causes any significant differences in the flocking transition.

From this discussion, it is obvious that a natural extension of our current work is interacting SPP models. If tumbling events are caused by cell-cell contacts, such a model would also allow us to predict how superdiffusivity changes with cell density. In even higher density cell populations and confluent tissues, cells will be in nearly constant contact and steric cell-cell interactions will play an even larger role in constraining cell positions. The effect of super-diffusion, whether generated by a Lévy walk or heterogeneity based model, could potentially alter the high-density behavior of standard SPP models.

For example, recent work suggests that groups of cells [36] and packings of SPPs undergo jamming transitions [11, 37, 38]. Could the addition of superdiffusive dynamics have an effect on these types of transitions? Persistent motility can alter the jamming transition—higher speeds and more persistent trajectories allows particles to explore areas of the energy landscape that were previously inaccessible [38]. Similar effects are seen in shape-based models for confluent tissues [36]. The inclusion of both run-and-tumble dynamics as well as varying persistence length through broadly distributed rotational diffusion coefficients in a generalized SPP model could offer an interesting mechanism for tuning jamming.

Another emergent feature of self-propelled particle models is motility induced phase separation (MIPS). Persistently moving particles create an inward oriented boundary layer that cage interior particles into a solid phase, while other cells are in a lower density gas phase outside of this boundary [37, 39] and this effect has recently been implicated in generating colony formation in bacteria [40]. MIPS relies on persistence length to generate this behavior. Our generalized SPP model could reinforce this effect due to relatively persistent run phases, destroy the effect due to tumbling, or perhaps alter the nature of the transition due to enhanced fluctuations, and this is an interesting direction for future study.

## Supporting information

S1 Text Cell and substrate preparation, imaging and analysis. (PDF)
