# Supplementary material for: Identifying the mechanism for superdiffusivity in mouse fibroblast motility
Source: PLoS Comput Biol. 2019 Feb 14;15(2):e1006732. doi: 10.1371/journal.pcbi.1006732 (PMC6392322; doi:10.1371/journal.pcbi.1006732)
Supplement: S1 Text — (PDF) [file pcbi.1006732.s001.pdf]

PNAS
www.pnas.org

Supplementary Information for

Identifying the mechanism for superdiffusivity in mouse fibroblast motility

Giuseppe Passucci, Megan E Brasch, James H Henderson, Vasily Zaburdaev, M Lisa Manning

Giuseppe Passucci.
E-mail: gpassucc@syr.edu

This PDF file includes:

Supplementary text
References for SI reference citations

Supporting Information Text

Cell and substrate preparation and imaging.. C3H10T1/2 mouse fibroblast cells (ATCC) were cultured in Basal Medium Eagle complete growth medium supplemented with 10% fetal bovine serum (v/v), 1% penicillin/streptomycin (v/v), and 1% GlutaMax (v/v). Cells were expanded in a 37°C humidified incubator with regulated 5% CO_2 and passaged at 80% confluence using 0.25% Trypsin EDTA. For time-lapse experiments, cells were restricted to passage numbers 12-18.

Although shape memory polymer substrates are typically used in cell culture to subject cells to dynamic topographies, in this work we focus on static, non-wrinkled SMP substrates. Poly(tert-butyl acrylate-co-butyl acrylate) (tBA:BA) SMP films were prepared as previously reported (1). Briefly, 95:5 wt% tBA:BA films were fabricated using 5 wt% tetraethylene glycol dimethacrylate as crosslinker and 0.06 wt% 2,2-dimethoxy-2-phenylacetophenone as a photoinitiator. Samples were cured for 30 minutes under UV light, followed by extraction in a 1:1 solution of methanol and distilled water overnight. Samples were then dried for at least 2 days in a 40°C vacuum oven prior to sample processing. Samples were cut into 6x6 mm squares and heated to 80°C on a hotplate to ensure no surface flaws were present. All samples were then sputter coated with gold for a total of 100 secs, resulting in an approximately 33 nm thick coating on the material surface. Samples were then UV sterilized for one hour on each side in a biological safety cabinet (ThermoFisher, 1300 Series A2) for subsequent cell culture.

Prior to cell seeding, SMP samples were soaked in room temperature BME medium for 6 hrs to promote fetal bovine serum protein adsorption to the material surface. Each sample was transferred into an individual well in a 48-well plate and cells were solution seeded (500 μL/well) at a density of 4000 cells/cm^2. Cell samples were then incubated at 30°C for 16 hrs to establish a relative equilibrium of cell motility prior to time-lapse image set-up. Hoechst nuclear stain was prepared at a concentration of 0.01 μg/mL in BME complete medium (30°C).

To stain and image, 800 μL of the staining solution was added to each well of a 4-well LabTek borosilicate chamber slide (Fisher Scientific) and incubated at 30°C for 1 hr. Samples were then inverted and weighed down with sterilized glass slide inserts, cut to fit into the chamber wells. The chamber slide was then transferred to a live cell stage incubator (INC-2000, 20/20 Technology, Inc.) and cells were imaged using a Leica DMI 6000B inverted microscope. The live-cell stage incubator was equilibrated at 30°C with constant 5% CO2. One image per position of interest was captured every five minutes in each of phase, A4 (excitation/emission peak of 360/470 nm), and N3 (excitation/emission peak of 546/600 nm) using 50 ms, 100 ms, and 50 ms exposure times respectively on an Andor Luca R camera with a 10x/0.63 NA objective. Samples were imaged in succession for 4 hrs at 30°C, followed by 20 hrs at 37°C. Although all of the data reported in this manuscript is from flat, control substrates, this temperature transition allowed other biocompatible SMP samples in the same chamber to transition to wrinkled nantopographies. We have shown previously that the temperature change alone does not change cell motility or polarization (1), which is also confirmed by the fact that the probability displacement distributions in our data set do not change from 5 to 20 hours, as shown in Fig 3(A) in the main text.

The resultant image stacks were analyzed using the ACTIVE image analysis package to track nuclei centers-of-mass (2).

Analyzing cell trajectories.. Cell motility was characterized using statistical analysis of cell nuclei trajectories. In general, the MSD for a collection of particle tracks is calculated as a function of time window (Δt):

$$MSD(\Delta t) = \frac{1}{N} \sum_{i=1}^{N} \sum_{t=1}^{T-\Delta t} [r_i(t + \Delta t) - r_i(t)]^2, \qquad [1]$$

where N is the total number of particles (cells), T is the total number of timesteps (frames), and $r_i(t)$ is the position of particle i at time t. An example of the MSD extracted from tracked mouse fibroblasts is shown in the main text in Fig. 2(A). As mentioned previously, the MSD can often be fit to the following functional form,

$$MSD(\Delta t) \propto \Delta t^{\alpha}. \qquad [2]$$

We extract the exponent α from data using a linear fit of $log_{10}(MSD)$ vs. $log_{10}(\Delta t)$. The dependence of fit on timescale Δt is extracted using a linear fit between timescales Δt and $\Delta t + T$ with $T = 100$. Altering the fitting window T did not significantly alter our results. Throughout the remainder of this manuscript we will denote the slope of the log-log MSD as α.

We calculate the standard directional velocity auto-correlation function:

$$C_{vv} = \langle \hat{v}(t_o)\hat{v}(t_o + dt)\rangle = \frac{\vec{v}(t_o)}{|\vec{v}(t_o)|} \frac{\vec{v}(t_o + dt)}{|\vec{v}(t_o + dt)|}, \qquad [3]$$

where the velocities, $\vec{v}(t)$, are the instantaneous displacements between two sequential frames and the brackets indicate averaging over the ensemble and initial times t_o. An example is shown in Fig. 1(B).

Unscaled displacement probability distributions were calculated by first constructing a cumulative distribution function (CDF) using the MATLAB function ksdensity. The probability distribution function is constructed as the numerical derivative of the CDF. This process was then repeated with scaled displacements $\rho(t) = \frac{r(t)}{t^{\gamma}}$ with the best collapse determined by minimizing the sum over the squared difference between each probability distribution and normalizing by the number of elements, sampling values of $0.4 < \gamma < 1$. Utilizing a bootstrap method of error estimation, the distributions from which the CDFs were constructed were randomly sampled with replacement to generate a new data set and the corresponding probability distribution. This process was iterated 100 times in order to estimate the variance of each bin.

A one dimensional Canny edge detection algorithm was used to calculate turning angle distributions (3). The input to this algorithm is a time series of changes in orientation, along with a threshold value. This differs from the traditional two dimensional Canny algorithm, used primarily to detect edges in images, and instead identifies "edges" in a one dimensional signal and labels them as either "runs" or "tumbles." The threshold is calculated using Otsu's method on individual cell trajectories (4), which uses a test angle magnitude, k, to divide the turning angles for a cell trajectory into two classes, runs and tumbles. The variance of each of these classes is then calculated as a function of k and minimized to determine the optimal threshold value for the Canny edge detection algorithm. The Canny edge detection algorithm then simply identifies changes in turning angle greater than the Otsu threshold as tumbling events. We define the run time as the time between tumbles. An example trajectory with highlighted tumbling events is shown in the main text in Fig. 1(B).

References

1. Yang P, Baker RM, Henderson JH, Mather PT. In vitro wrinkle formation via shape memory dynamically aligns adherent cells. Soft Matter. 2013;9:4705. doi:10.1039/c3sm00024a.
2. Baker RM, Brasch ME, Manning ML, Henderson JH. Automated, contour-based tracking and analysis of cell behaviour over long time scales in environments of varying complexity and cell density. Journal of the Royal Society, Interface / the Royal Society. 2014;11(97):20140386. doi:10.1098/rsif.2014.0386.
3. Canny J. A Computational Approach to Edge Detection. IEEE Transactions on Pattern Analysis and Machine Intelligence. 1986;PAMI-8(6):679–698. doi:10.1109/TPAMI.1986.4767851.
4. Otsu N. A threshold selection method from gray-level histograms. IEEE Transactions on Systems, Man, and Cybernetics. 1979;9(1):62–66. doi:10.1109/TSMC.1979.4310076.
